# Variability in pelvic packing practices for hemodynamically unstable pelvic fractures at US level 1 trauma centers

**DOI:** 10.1186/s13037-019-0183-7

**Published:** 2019-01-16

**Authors:** Benoit Blondeau, Alessandro Orlando, Stephanie Jarvis, Kaysie Banton, Gina M. Berg, Nimesh Patel, Rick Meinig, Allen Tanner, Matthew Carrick, David Bar-Or

**Affiliations:** 1 Research Medical Center, 2316 East Meyer Blvd., Kansas City, MO 64132 USA; 2 University of Connecticut, Hartford Hospital, Hartford, CT 06106 USA; 3 Trauma Research, LLC., 383 Corona St. #319, Denver, CO 80218 USA; 4 Swedish Medical Center, 501 E Hampden Ave, Englewood, CO 80113 USA; 5 Wesley Medical Center, 550 N. Hillside St, Wichita, KS 67214 USA; 6 St. Anthony’s Hospital, 11600 West 2nd Place, Lakewood, CO 80228 USA; 7 Penrose Hospital, 2222 North Nevada Ave, Colorado Springs, CO 80907 USA; 8 Medical City Plano, 3901 West 15th Street, Plano, TX 75075 USA

**Keywords:** Pelvic packing, Pelvic fracture management, Trauma, Level 1 trauma centers, Trauma medical directors, Survey

## Abstract

**Background:**

Mortality from hemodynamically unstable pelvic fractures remains high. Guidelines offer varying care approaches including the use of pelvic packing (PP), which was recently adopted for potential control of bleeding for this condition. However, the implementation of PP is uncertain as the debate on the optimal resuscitation strategy, angioembolization or PP continues. The study was designed to assess current practices among level 1 trauma centers in the US in regard to PP treatment for hemodynamically unstable pelvic fractures.

**Methods:**

A cross-sectional survey was created to assess when to apply PP, application approach, and the respondent’s anecdotal perception on safety and effectiveness. Trauma Medical Directors at 158 US level 1 trauma centers were sent biweekly email invitations for 3 months. Participants were allowed to skip questions for any reason. The study hypothesis was that PP practices vary by US census bureau region, annual trauma admissions, and length of time in years since each trauma center received their respective level 1 trauma center designation.

**Results:**

Twenty-five percent (40/158) of trauma medical directors participated and 75% (118/158) of the trauma medical directors did not participate. Of those who took the survey, 36/40 (90%) completed the survey and 4/40 (10%) partially completed the survey. Only 36 trauma medical directors responded on their perception of safety and effectiveness; 72% (26/36) of participants perceived PP as safe, whereas only a third (12/36) of participants perceived PP as effective. There were 25 trauma medical directors who provided the sequence of treatment modalities utilized at their level 1 trauma center, 76% (19/25) of participants reported that PP is utilized as the third or fourth priority. None of the participating level 1 trauma centers reported a preference towards utilization of PP as the first priority treatment. Half of the participants reported a preference towards applying PP only as a last resort to control hemorrhage. Northeastern and Western level 1 trauma centers were significantly more likely than Midwestern and Southern level 1 trauma centers to have reported application of PP to all hemodynamically unstable patients (*p* = 0.05). Midwestern, Southern, and Western level 1 trauma centers were significantly more likely to have perceived PP as safe than Northeastern level 1 trauma centers (*p* = 0.04). All low-volume and 38% high-volume level 1 trauma centers perceived PP to increase infection risks, (*p* = 0.03). We observed no association between the length of time each trauma center was designated a level 1 trauma center, and all participant responses.

**Conclusion:**

Controversy and varying anecdotal perception regarding safety and effectiveness of PP prevails among trauma medical directors at level 1 trauma centers in the US.

## Background

For a hemodynamically unstable patient with a pelvic fracture, the use of preperitoneal or retroperitoneal pelvic packing (PP) is one of the more controversial treatment methods and is evidenced by varying recommendations in guidelines. The Advanced Trauma Life Support guideline [1] does not include the application of PP, whereas the World Society of Emergency Surgeons (WSES) [2] states all patients who are hemodynamically unstable should have preperitoneal PP considered for placement first, prior to any other intervention. Evidence of further incongruity is in the Eastern Association for the Surgery of Trauma (EAST) [3] recommendations, where it suggests the use of retroperitoneal PP only after angiographic embolization, or as part of a multidisciplinary approach with a pelvic orthotic device. Similarly, Western Trauma Association (WTA) [4] recommends applying preperitoneal PP after angiography or angioembolization if the patient remains hemodynamically unstable and after a negative focused assessment with sonography for trauma (FAST) with consideration for placement of an external fixator. Lastly, the Trauma Quality Improvement Program (TQIP) [5] guideline states that preperitoneal PP should be used when angiography is unavailable, as well as after a negative FAST, preferably following the application of an external fixation device. Although Cothren et al. [6] described the development of pelvic packing as a “paradigm shift,” there is little consensus and the recommendations for PP vary by organization and by region.

Owing to the diverse guidelines for the use of PP, the purpose of this study was threefold, to describe: 1) hospital practices on the use of PP at American College of Surgeons (ACS) verified Level 1 trauma centers in the United States, 2) the beliefs of the trauma medical directors on the safety and efficacy of PP, 3) how PP preferences varied by region, annual trauma admissions, and length of time in years as an ACS-verified level 1 trauma center. The study hypothesis was that management practices would vary by region, annual trauma admissions, and years as a level 1 trauma center.

## Methods

### Study sites

This study was approved by the Western Institutional Review Board. This was a cross-sectional survey of trauma medical directors at ACS-verified level 1 trauma centers in the United States. Per ACS guidelines, [7] level 1 trauma centers must admit at least 1200 trauma patients annually or must have 240 trauma admissions with an injury severity score of more than 15. We derived a survey invitation list of 158 US ACS-verified level 1 trauma centers using the ACS website, which outlined all ACS-verified level 1 trauma centers by state; to view a list of the level 1 trauma centers invited to participate please visit: http://bit.ly/TraumaCenterInvites. Names and contact information for the trauma medical director at each identified level 1 trauma center were attained via facility websites or personal communication. If the trauma center had a vacant trauma medical director position, the trauma center was called to determine who was acting as the interim trauma medical director for the survey invitation.

We sent email invitations via SurveyMonkey Inc. (San Mateo, California; www.surveymonkey.com) containing the approved consent form with a partial waiver of consent that required no signature. The consent form included directions on how to participate or decline participation for the web-based survey on SurveyMonkey. Survey participation was voluntary, and no compensation was provided. Either the trauma medical director, or an assigned colleague, completed the survey. Trauma medical directors are referred to as “participants.”

### Survey methods

Survey questions and responses were drafted using the online survey tool SurveyMonkey. Co-authors revised the questions and two trauma medical directors piloted the survey. SurveyMonkey’s feature ‘skip logic’ skipped irrelevant questions based on previous responses. Participants could skip any additional questions for any reason. Coauthors approved the survey’s final draft, and the survey was sent to trauma medical directors via email with a link to access the web-based survey through SurveyMonkey. The survey included 46 questions, of which 15 are relevant to this study; to view these questions and possible choices, please visit: http://bit.ly/PelvicPackingSurvey. Participants were asked questions regarding trauma center demographics characteristics such as: the US Census Bureau [8] region that the trauma center resides, the volume of annual trauma admissions, and the length of time in years the trauma center has been designated an ACS-verified level 1 trauma center. Participants were also asked about PP preferences including: when PP was applied, who PP was applied to, what approach was used for application, as well as their opinion regarding safety and effectiveness.

After the initial invitation, five survey reminders were sent every 2 weeks to trauma medical directors who had not responded nor declined participation. Survey invitations and reminders were sent from March 1, 2018 to June 26, 2018. SurveyMonkey tracked participation while keeping responses anonymous to reduce potential bias. If the trauma medical director did not respond nor decline by the fifth reminder and final survey reminder, we called the trauma medical director to inform them of the study and verify they received the invitation.

The primary outcome was the use of PP. Secondary outcomes included: type of PP used (retroperitoneal, preperitoneal, or both), who PP was applied to (all hemodynamically unstable patients or hemodynamically stable patients), when PP was applied (open ended question), if PP was used only as a last resort to control hemorrhage, perception on the safety of PP, perception on the effectiveness of PP, perception on the infection risks of PP, and lastly perception on the benefits of PP.

Survey responses were extracted from SurveyMonkey and SAS 9.4 (Cary, NC) software was used for analysis. Descriptive data were expressed as numbers or proportions. Statistical differences in categorical variables such as region that the trauma center resides, the volume of annual trauma admissions, and the length of time in years as an ACS-verified level 1 trauma center were assessed using Fisher’s exact test. An alpha value of 0.05 was used.

## Results

There were 40/158 survey responses, resulting in a response rate of 25%. Of those, 36/40 (90%) completed the survey and 4/40 (10%) partially completed the survey. There were 118/158 (75%) trauma medical directors who did not respond to survey. The partially completed responses were included in the final analysis. The survey took a median (IQR) of 11 min (8-21) to complete.

Table 1 summarizes the trauma center demographics. The most common response for the U.S. Census Bureau region was the South, 40% (16/40), followed by the Midwest, 25% (10/40), the Northeast, 20% (8/40), and the West, 15% (6/40). Ninety percent (36/40) of the participating level 1 trauma centers had a high volume of trauma admissions, defined as > 1500 patients in 2017. Ten percent (4/40) of the participating trauma centers had a low volume of trauma admissions, defined as ≤ 1500 patients admitted in 2017. The majority, 58% (23/40), of participating level 1 trauma centers had been an ACS-verified level 1 trauma center for over 10 years.

**Table 1:** Survey responses

Question	Response Option	Results % (n)	n
U.S Census Bureau Region	Midwest	25% (10)	40
	Northeast	20% (8)	
	South	40% (16)	
	West	15% (6)	
Number of 2017 trauma admissions	Low-Volume ≤ 1500	10% (4)	40
	High-Volume 1501–4000	90% (36)	
Length of time as Level 1 Trauma Center	< 1 year	5% (2)	40
	> 1 to 2 years	15% (6)	
	> 2 to 5 years	18% (7)	
	> 5 to 10 years	5% (2)	
	> 10 years	58% (23)	
Organization that developed guideline	EAST	43% (9)	21
	WTA	29% (6)	
	TQIP	14% (3)	
	ATLS	10% (2)	
	Other	5% (1)	
	WSES	0	
Pelvic packing used	Yes	83% (30)	36
	No	17% (6)	
Indicators for pelvic packing	Hemodynamically Unstable	34% (10)	29
	After Ex-Lap	10% (3)	
	After angiography	3% (1)	
	No blush, unstable after Angio	7% (2)	
	IR Unavailable	17% (5)	
	In OR	3% (1)	
	Increasing hematoma in OR	3% (1)	
	Last resort	10% (3)	
	Physicians judgement	10% (3)	
Type of pelvic packing used	Retroperitoneal	3% (1)	30
	Preperitoneal	53% (16)	
	Both	43% (13)	
Pelvic packing used only as a last resort	Yes	47% (14)	30
	No	53% (16)	
Pelvic packing used on all hemodynamically unstable patients	Yes	13% (2)	16
	No	88% (14)	
Pelvic packing is a treatment option for hemodynamically stable patients	Yes	6% (1)	16
	Sometimes	50% (8)	
	No	44% (7)	
Pelvic packing is a safe treatment method	Yes	72% (26)	36
	Sometimes	25% (9)	
	No	3% (1)	
Pelvic packing is an effective treatment method	Yes	33% (12)	36
	Sometimes	64% (23)	
	No	3% (1)	
Pelvic packing increases risk for infection	Yes	44% (16)	36
	No	56% (20)	
The benefits of pelvic packing outweigh the risks	Yes	94% (15)	16
	No	6% (1)	

*WTA* Western Trauma Association, *EAST* Eastern Association for Surgery and Trauma, *TQIP* Trauma Quality Improvement Program, *WSES* World Society of Emergency Surgeons, *ATLS* Advanced Trauma Life Support, *Ex-Lap* Exploratory Laparotomy, *Angio* Angiography, *IR* Interventional Radiology, *OR* Operating Room

The top two guidelines followed were: EAST, 43% (9/21) and WTA, 29% (6/21). No participants followed the WSES guideline. Pelvic packing was reported as a treatment method utilized at 83% (30/36) of the level 1 trauma centers. Only 3 % (1/36) of level 1 trauma centers utilized solely retroperitoneal PP, 53% (16/30) used preperitoneal PP only, and 43% (13/30) used both retroperitoneal and preperitoneal PP.

Trauma medical directors were asked their anecdotal beliefs on the safety, effectiveness, risk of infection, and if the benefits of PP outweighed the risks for infection. The majority of participants, 72% (26/36), perceived that PP was a “safe” treatment method, 25% (9/36) perceived that PP was “sometimes safe”, and one participant perceived that PP was “not safe”. With regard to the perceived effectiveness of PP, 33% (12/36) of the participants perceived that PP was “effective”, 64% (23/36) of participants perceived that PP was “sometimes” an effective treatment method and one perceived that PP was “not effective”. Participants were split when asked if PP increased the risk for infection, 44% (16/36) of participants perceived PP increased the risks for infection and 56% (20/36) perceived PP did not increase the risks for infection. Only one participant did not perceive that the benefits of PP outweighed the risks for infection.

Participants were queried to determine the priority of each treatment management technique for hemodynamically unstable patients; Table 2 summarizes their responses. Although there was strong agreement reported on a preference towards utilization of circumferential compression devices as the first priority treatment method, there was less agreement on the second priority treatment method; there was a near three-way tie between the use of resuscitative endovascular balloon occlusion of the aorta (REBOA), angiography, and exploratory laparotomy. Most of the participants reported that their trauma center utilized PP as the third 36% (9/25,) or fourth 40% (10/25) priority treatment method. Exploratory laparotomy and external fixation device were reported high as the fifth priority treatment utilized for hemodynamically unstable pelvic fractures. Finally, REBOA was the most reported sixth priority treatment utilized.

**Table 2:** Sequence of treatment methods for pelvic fracture management

Treatment Option	Sequence of Treatment Method					
	First	Second	Third	Fourth	Fifth	Sixth
CCD	89%	0%	7%	4%	0%	0%
REBOA	0%	33%	13%	20%	7%	27%
PP	0%	12%	36%	40%	8%	4%
Angiography	11%	30%	30%	22%	4%	4%
Exploratory Laparotomy	0%	30%	15%	5%	35%	15%
External Fixation Device	0%	22%	13%	22%	30%	13%

Participants were asked to denote the sequence of treatment for a hemodynamically unstable patient with a pelvic fracture using the options provided. *CCD* Circumferential Compression Device, *REBOA* Resuscitative Endovascular Balloon Occlusion of the Aorta, *PP* Pelvic Packing

Notwithstanding the consensus on the reported application of PP as a middle priority treatment for hemodynamically unstable pelvic fractures, the indications for its use varied (Table 1). The two most common indications for PP were hemodynamic instability and the unavailability of interventional radiology.

The application of PP to all hemodynamically unstable patients significantly differed by region, Table 3. Northeast and West level 1 trauma centers were more likely than Midwest and South level 1 trauma centers to report that their trauma center applied PP to all hemodynamically unstable pelvic fractures (*p* = 0.05). Midwest, South, and West level 1 trauma centers were more likely to perceive that PP was “safe” or “sometimes safe”, whereas Northeast level 1 trauma centers were more likely to perceive that PP was “sometimes safe” or “not safe” (*p* = 0.04). Although more regions perceived PP as more safe than unsafe, there was a less positive response when asked if PP was effective. In every region, the number of participants responding that PP was safe was double that when asked if PP is effective.

**Table 3:** Regional analysis of pelvic packing practices

	Midwest	Northeast	South	West	n	p
Is PP used?						
Yes	80% (8/10)	57% (4/7)	92% (12/13)	100% (6)	36	0.18
No	20% (2/10)	43% (3/7)	8% (1/13)	0% (0)		
Type of PP used						
Use PPP only	75% (6/8)	50% (2/4)	33% (4/12)	667% (4/6)	30	0.16
Use RPP only	13% (1/8)	0% (0)	0% (0)	0% (0)		
Use both	13% (1/8)	50% (2/4)	67% (8/12)	33% (2/6)		
Use PP only as a last resort						
Yes	38% (3/8)	75% (3/4)	42% (5/12)	50% (3/6)	30	0.75
No	63% (5/8)	25% (1/4)	58% (7/12)	50% (3/6)		
Apply PP to ALL hemodynamically Unstable Patients						
Yes	0% (0)	100% (1)	0% (0)	33% (1/3)	16	0.05
No	100% (5)	0% (0)	100% (7)	67% (2/3)		
PP is an option for hemodynamically Stable Patients						
Yes	0% (0)	0% (0)	15% (1/7)	0% (0)	16	0.10
Sometimes	40% (2/5)	100% (1)	71% (5/7)	0% (0)		
No	60% (3/5)	0% (0)	14% (1/7)	100% (3)		
PP is safe						
Yes	80% (8/10)	29% (2/7)	92% (12/13)	67% (4/6)	36	0.04
Sometimes	20% (2/10)	57% (4/7)	8% (1/13)	33% (2/6)		
No	0% (0)	14% (1/7)	0% (0)	0% (0)		
PP is effective						
Yes	40% (4/10)	14% (1/7)	38% (5/13)	33% (2/6)	36	0.68
Sometimes	60% (6/10)	71% (5/7)	62% (8/13)	67% (4/6)		
No	0% (0)	14% (1/7)	0% (0)	0% (0)		
PP increases the risk for infection						
Yes	50% (5/10)	44% (3/7)	23% (3/13)	83% (5/6)	36	0.11
No	50% (5/10)	57% (4/7)	77% (10/13)	17% (1/6)		
PP benefits outweigh the risks						
Yes	80% (4/5)	100% (3)	100% (3)	100% (5)	16	0.99
No	20% (1/5)	0% (0)	0% (0)	0% (0)		

Regions were defined using the U.S. Census Bureau’s definition. *PP* Pelvic Packing, *RPP* Retroperitoneal Pelvic Packing, *PPP* Preperitoneal Pelvic Packing

The relationship between 2017 volume of trauma admissions and PP practices can be seen in Table 4. Seventy-five percent (3/4) of the low-volume level 1 trauma centers utilized PP as a treatment modality and 84% (27/32) of the high-volume level 1 trauma centers utilized PP (*p* = 0.53). All the low-volume level 1 trauma centers that used PP, applied PP only as a last resort to control hemorrhage (4/4); whereas of the high-volume level 1 trauma centers that use PP, 41% (11/27) utilized PP only as a last resort to control hemorrhage (*p* = 0.09). All the low-volume trauma center’s participants perceived that PP placed the patient at an increased risk for infection, whereas 38% (12/32) high-volume trauma center’s participants perceived that PP placed the patient at an increased risk for infection (*p* = 0.03). However, all the participants at low-volume level 1 trauma centers and 92% (11/12) of the participants at high-volume level 1 trauma centers perceived that the benefits of PP outweighed the risk for infection.

**Table 4:** Analysis of hospital volume and pelvic packing practices

	High-volume > 1500 admissions	Low-volume < 1500 admissions	n	p
Is PP used?				
Yes	84% (27/32)	75% (3/4)	36	0.53
No	16% (5/32)	25% (1/4)		
Type of PP used				
Use PPP only	56% (15/27)	33% (1/3)	30	0.62
Use RPP only	3.7% (1/27)	0% (0)		
Use both	41% (11/27)	67% (2/3)		
Use PP only as a last resort				
Yes	41% (11/27)	100% (3)	30	0.09
No	59% (16/27)	0% (0)		
Apply PP to ALL hemodynamically unstable patients				
Yes	12% (2/16)	0% (0)	16	N/A
No	88% (14/16)	0% (0)		
PP is an option for hemodynamically stable patients				
Yes	6% (1/16)	0% (0)	16	N/A
Sometimes	50% (8/16)	0% (0)		
No	44% (7/16)	0% (0)		
PP is safe				
Yes	75% (24/32)	50% (2/4)	36	0.35
Sometimes	22% (7/32)	50% (2/4)		
No	3% (1/32)	0% (0)		
PP is effective				
Yes	38% (12/32)	0% (0)	36	0.36
Sometimes	59% (19/32)	100% (4)		
No	3% (1/32)	0% (0)		
PP increases the risk for infection				
Yes	38% (12/32)	100% (4)	36	0.03
No	63% (20/32)	0% (0)		
PP benefits outweigh the risks				
Yes	92% (11/12)	100% (4)	16	0.99
No	8% (1/12)	0% (0)		

*PP* Pelvic Packing, *RPP* Retroperitoneal Pelvic Packing, *PPP* Preperitoneal Pelvic Packing

Table 5 shows how PP practices varied across level 1 trauma centers when categorized by the length of time the trauma center has been designated an ACS-verified Level 1 trauma center. Two (18.18%) level 1 trauma centers that had been designated a level 1 trauma center for ≥10 years reported a preference towards application of PP to all hemodynamically unstable patients, no other level 1 trauma centers reported a preference towards application of PP to all hemodynamically unstable patients. Overall, we observed a lack of significant association between the length of time since the participating level 1 trauma centers received their respective level 1 trauma center designation and all participant responses.

**Table 5:** Pelvic packing and length of time in years as an ACS-verified level 1 trauma center

	< 1 Year	> 1 to 2 Years	> 2 to 5 Years	> 5 to 10 Years	> 10 Years	n	p
PP is used							
Yes	100% (2)	60% (3/5)	71% (5/7)	50% (1/2)	95% (19/20)	36	0.10
No	0% (0)	40% (2/5)	29% (2/7)	50% (1/2)	5% (1/20)		
Type of PP used							
PPP only	50% (1/2)	67% (2/3)	60% (3/5)	0% (0)	53% (10/19)	30	0.97
RPP only	0% (0)	0% (0)	0% (0)	0% (0)	5% (1/19)		
Both	50% (1/2)	33% (1/3)	40% (2/5)	100% (1)	42% (8/19)		
Use PP only as a last resort							
Yes	50% (1/2)	33% (1/3)	60% (3/5)	100% (1)	42% (8/19)	30	0.85
No	50% (1/2)	67% (2/3)	40% (2/5)	0% (0)	58% (11/19)		
Apply PP to all hemodynamic unstable patients							
Yes	0% (0)	0% (0)	0% (0)	0% (0)	18% (2/11)	16	0.99
No	100% (1)	100% (2)	100% (2)	0% (0)	82% (9/11)		
PP is an option for hemodynamic stable patients							
Yes	0% (0)	0% (0)	0% (0)	0% (0)	9% (1/11)	16	0.8
Sometimes	0% (0)	100% (2)	50% (1/2)	0% (0)	45% (5/11)		
No	100% (1)	0% (0)	50% (1/2)	0% (0)	45% (5/11)		
PP is safe							
Yes	100% (2)	80% (4/5)	71% (5/7)	0% (0)	75% (15/20)	36	0.2
Sometimes	0% (0)	20% (1/5)	14% (1/7)	100% (2)	25% (5/20)		
No	0% (0)	0% (0)	14% (1/7)	0% (0)	0% (0)		
PP is effective							
Yes	0% (0)	40% (2/5)	14% (1/7)	0% (0)	45% (9/20)	36	0.44
Sometimes	100% (2)	60% (3/5)	71% (5/7)	100% (2)	55% (11/20)		
No	0% (0)	0% (0)	14% (1/7)	0% (0)	0% (0)		
PP increases the risk of infection							
Yes	100% (2)	20% (1/5)	43% (3/7)	50% (1/2)	45% (9/20)	36	0.57
No	0% (0)	80% (4/5)	57% (4/7)	50% (1/2)	55% (11/20)		
PP benefits outweigh the risks							
Yes	100% (2)	100% (1)	67% (2/3)	100% (1)	100% (9)	16	0.44
No	0% (0)	0% (0)	33% (1/3)	0% (0)	0% (0)		

*PP* Pelvic Packing, *RPP* Retroperitoneal Pelvic Packing, *PPP* Preperitoneal Pelvic Packing

## Discussion

Most published guidelines recommend the use of PP for hemodynamically unstable patients. However, when following the WTA [4] algorithm, PP is also an option for hemodynamically stable patients in two scenarios: 1) after a pelvic blush is seen during angiography and unsuccessful angioembolization or 2) for a hemodynamically stable patient who is admitted to the ICU and then has an unsuccessful angioembolization. One participant responded that PP is “an option” for hemodynamically stable patients and seven participants surveyed stated that PP is “sometimes an option” for hemodynamically stable patients but none of those participants were following the WTA guideline. Since the early description of PP application, it has conventionally been applied to hemodynamically unstable patients, typically in extremis.

When PP was first used, it was applied for damage control using the transperitoneal approach by making an incision in the intact peritoneum disrupting the pelvic hematoma, and it was commonly used as a last course of resuscitation in patients who were coagulopathic [9–12]. These practices resulted in poor patient outcomes and many physicians abandoned the use of PP. The findings of this study indicate that roughly half of participants applied PP only as a last resort for hemorrhage control. This may explain why 67% of participants perceived that PP is “sometimes” or “not effective”. The techniques for PP application have since evolved and currently no guidelines recommend the application of PP as a last resort to control hemorrhage. In fact, the WSES [2] states that all hemodynamically unstable patients should have PP considered prior to any other intervention.

There is a lack of consensus in the literature on when to apply PP and the sequence of treatment methods; this is reflected by the variation in survey responses. The most common indicator for PP reported was hemodynamic instability, yet only two participants reported that PP is applied to all hemodynamically unstable patients at their trauma center. Whereas, the WSES [2] guideline recommends considering PP as the first-line of treatment for all hemodynamically unstable patients. A majority of participating level 1 trauma centers reported PP was utilized as the third or fourth priority treatment for hemodynamically unstable pelvic fractures. The TQIP [5] recommends to apply PP in two different scenarios for hemodynamically unstable patients, as the second or third priority treatment. The WTA [4] also applies PP in two scenarios for hemodynamically unstable patients, as the second or third priority treatment. The EAST [3] guideline uses PP as the third priority treatment. The guidelines recommend earlier application of PP, as the first, second, or third priority treatment; whereas our survey results show that level 1 trauma centers applied PP later in the treatment course, as the third or fourth priority treatment. For more information on the guideline’s treatment sequence see Table 6.

**Table 6:** Sequence of treatment methods in published guidelines for hemodynamically unstable patients

	WSES	TQIP *FAST +*	TQIP *Fast -*	TQIP *Extremis*^*a*^	WTA *Fast -*	WTA *FAST +*	EAST	ATLS
First	PP	CCD	CCD	REBOA	CCD	CCD	CCD	CCD
Second	CCD, REBOA, and / or Angio	Ex-Lap	Ex-Fix	PP	Ex-Fix, REBOA, or PP	Ex-Lap & Ex-Fix	Angio	Ex-Lap or Angio
Third	Ex-Lap	Consider Ex-fix	PP	Ex-Lap	PP or Angio	PP	PP	Ex-Fix
Fourth	Re-angio		Angio	Ex-Fix	Angio	Angio		
Fifth	Ex-Fix			Angio				

*WSES* World Society of Emergency Surgeons, *TQIP* Trauma Quality Improvement Program, *FAST* Focused assessment with sonography, *WTA* Western Trauma Association, *EAST* Eastern Association for Surgery and Trauma, *ATLS* Advanced trauma life support, *PP* Pelvic Packing, *CCD* Circumferential compression device, *REBOA* Resuscitative endovascular balloon occlusion of the aorta, *Ex-Lap* Exploratory Laparotomy, *Ex-Fix* External fixation device, *Angio* Angiography with embolization if indicated. ^a^In extremis solely from pelvic bleeding

Among the ranked second-line of treatment methods appeared REBOA. Brenner et al. [13] issued a joint policy statement addressing the current practices of REBOA. They stated there is currently no high-grade evidence that demonstrates REBOA improved outcomes or survival compared to standard treatment of severe hemorrhage. In addition, they state that the benefits of REBOA are unproven. Despite this, most of those that reported their trauma center utilized REBOA, utilized it earlier than other approaches for hemodynamically unstable pelvic fractures. In 2017, Stahel et al. [14] conducted a literature review and reported that REBOA could effectively bridge the time to the operating room until definitive surgical bleeding control, but that future validation studies are needed to determine its use in the multidisciplinary management of pelvic fractures.

Most of the guidelines recommending the application of PP, state to use the *preperitoneal* method; only the EAST guideline [3] recommends the use of the *retroperitoneal* method. However, within the EAST guideline’s scientific foundation section for *retroperitoneal* PP, EAST ironically only includes literature on *preperitoneal* PP. This may be the cause of the variations in practice in the current findings. Although the most common response for what guideline the trauma center was following was the EAST guideline, only a couple of level 1 trauma centers following the EAST guideline stated they used both retroperitoneal and preperitoneal PP, and none stated they used solely retroperitoneal PP. Smith et al. [15] described the surgical technique for *retroperitoneal* pelvic packing in 2005. They state during the *retroperitoneal* approach, an 8-cm midline incision from the symphysis pubis in the caudal direction is made, skin and subcutaneous tissue are incised, and the fascia is divided in the midline. The pelvic brim is palpated, and laparotomy sponges are placed as deep to the brim as possible in the true pelvis. In 2007, Cothren et al. [6] published the first epidemiological study assessing outcomes in patients after modifying the technique for PP application by transitioning from applying PP to the *retroperitoneum*, to directly packing the pelvis using a *preperitoneal* approach addressing venous and bone hemorrhage without disrupting the pelvic hematoma. They described the surgical technique for the *preperitoneal* approach as a 6- to 8-cm midline incision made from the public symphysis in the cephalad direction. The midline fascia is also divided using the *preperitoneal* approach, but the peritoneum is left intact. *Preperitoneal* PP is applied to each side of the bladder into the pelvis below the pelvic brim. The Cothren et al. article on *preperitoneal* PP, as well as other articles on *preperitoneal* PP, were cited in the EAST guideline as evidence for application of *retroperitoneal* pelvic packing.

The safety and effectiveness of PP remains controversial; there was incongruence in participant’s responses when asked if PP was an effective treatment method to control pelvic hemorrhage. Only a third of participants perceived that PP was effective. Marzi et al. [16] conducted a literature review on pelvic fracture management in 2009 and concluded that pelvic packing should be used for hemodynamically unstable patients who do not respond to resuscitative fluids and those requiring continuous fluids to maintain hemodynamic stability. Many studies [9, 11, 16–18] have compared PP to angioembolization as a treatment modality, leading to some [4, 15, 19–21] concluding that that the two approaches should not be compared and should instead be viewed as complementary. In 2018, Muntasar et al. [9] found insufficient evidence to support superiority of one treatment modality over the other. Cothren et al. [6] observed a reduction in their own mortality rate in patients with pelvic fractures, from roughly 50 to 25%, after adopting an algorithm using PP. Although, trauma medical directors did not agree on the effectiveness of PP, a majority of the trauma medical directors perceived that PP was a “safe” treatment method or “sometimes safe”; only one trauma medical director perceived that PP was “not safe”.

The main complication of PP reported is pelvic space infections, which may be a contributing factor to the finding that over a quarter of participants perceived that PP was “not safe” or “sometimes safe”. Burlew et al. found [19] that 6% of patients with PP developed infections, and 47% of patients who required repacking developed pelvic space infections. In another study conducted by Burlew et al. [21], 12% of patients with preperitoneal PP developed pelvic space infections. Totterman et al. [12] reported a pelvic infection rate of 33% after PP. None of these studies compared infection rates between PP and other pelvic fracture treatment modalities. Li et al. [17] and Osborn et al. [22] compared the infection rates between patients who had PP applied to patients who had angiography or angioembolization and found no significant difference in infection rates. Our findings indicate a lack of consensus on participants’ beliefs regarding the risk for infection. Approximately half of the trauma medical directors perceived that PP increased the risks for infection, only one trauma medical director perceived that the benefits of PP did not outweigh the risks of infection.

### Limitations

The results of this study are limited by the response rate of 25% (40/158); there were 118/158 (75%) trauma medical directors who did not participate. Contacting the participants proved difficult as contact information was not always accurate. This resulted in delayed contact with the participants, fewer reminders being sent, and a shortened response time. The survey responses represented the perspective of the responding trauma medical director and were not independently verified. Trauma medical directors’ responses regarding current protocols and guidelines may have been based on their memory, not referring to actual trauma center protocols, and subject to biases such as selective memory, telescoping, attribution, and exaggeration. The survey instructions informed trauma medical directors to have the guideline or protocol on-hand twice, in the invitation and upon opening the survey, to reduce the effect of self-reported data bias. Although the survey was anonymous, the study could reveal a level of inefficiency and unpreparedness in face of complex trauma patients, further limiting the response rate. Survey accessibility was also a limitation. The study protocol was approved for SurveyMonkey® distribution, limiting those who preferred paper surveys, noted in reminder phone calls. One trauma medical director did not receive the survey due to hospital server restrictions.

## Conclusions

The treatment of hemodynamically unstable patients with pelvic fractures is a complex management strategy. The WTA, EAST, TQIP, ATLS, and WSES [1–5] guidelines offer varying approaches to care for patients with pelvic fractures, with PP application having the least consistency across guidelines. As such, current preferences on use of PP vary across the United States at ACS-verified level 1 trauma centers. The sequence of treatment methods is disputed in the literature, which is reflected by our findings on the sequence of treatment modalities. Guidelines recommend earlier application of PP than found in our survey; almost half of the level 1 trauma centers reported a preference towards PP application only as a last resort to control hemorrhage when none of the guidelines recommend PP to be applied as a last resort. Level 1 trauma centers should re-evaluate their pelvic fracture treatment protocols and their interpretation of published guidelines to ensure utilization of protocols based on the most recent findings. Not only is the sequence of treatment methods controversial, but also who should have PP applied. Survey results show that that the most common indicator for PP reported by participants was hemodynamic instability, however few level 1 trauma centers applied PP to all hemodynamically unstable patients with a pelvic fracture. Although a majority of trauma medical directors perceived that PP was a safe treatment modality, only a third of participants perceived that PP was effective. There is a need for research studies that determine the optimal time to apply PP. Studies comparing the effectiveness of varying treatment sequences, rather than simply comparing outcomes by treatment modality, could substantially add to the literature available and impact the guidelines available on pelvic fracture management.

## Abbreviations

ACS: American College of Surgeons
EAST: Eastern Association for the Surgery of Trauma
FAST: Focused assessment with sonography
PP: Pelvic Packing
REBOA: Resuscitative Endovascular Balloon Occlusion of the Aorta
TQIP: Trauma Quality Improvement Program
WSES: World Society of Emergency Surgeons
WTA: Western Trauma Association
